# Genome-wide analysis of Epstein-Barr virus (EBV) isolated from EBV-associated gastric carcinoma (EBVaGC)

**DOI:** 10.18632/oncotarget.6751

**Published:** 2015-12-24

**Authors:** Ying Liu, Wenjun Yang, Yaqi Pan, Jiafu Ji, Zheming Lu, Yang Ke

**Affiliations:** ^1^ Key Laboratory of Carcinogenesis and Translational Research (Ministry of Education), Laboratory of Genetics, Peking University Cancer Hospital and Institute, Haidian, Beijing, China; ^2^ Key Laboratory of Reproduction and Heredity of Ningxia Region, Medical Oncology Department of General Hospital, Ningxia Medical University, Yinchuan, Ningxia, China; ^3^ Key Laboratory of Carcinogenesis and Translational Research (Ministry of Education), Department of Gastrointestinal Surgery, Peking University Cancer Hospital and Institute, Haidian, Beijing, China

**Keywords:** Epstein-Barr virus, gastric carcinoma, EBVaGC, next-generation sequencing

## Abstract

Epstein-Barr virus (EBV) is linked to the development of a variety of malignancies, including EBV-associated gastric carcinoma (EBVaGC). In this study, EBVaGC was detected in 15 (7.3%) of 206 GC cases. To identify the EBV genomic variation, EBV genomic sequences isolated from 9 EBVaGC biopsy specimens were successfully retrieved, designated EBVaGC1 to EBVaGC9. By comparative analysis of these strains with another 6 completely sequenced EBV strains, EBV-wild type, B95–8, AG876, GD1, GD2, and HKNPC1, it was demonstrated that EBVaGC1 to 9 were most closely related to the GD1 strain. Phylogenetic analysis of the GC biopsy specimen-derived EBV (GC-EBV) genomes was subsequently performed to assess their genomic diversity and it exhibited the greatest divergence from the type 2 strain, AG876. Compared with the reference EBV strain GD1, they harbored 961 variations in total, including 919 substitutions, 23 insertions, and 19 deletions. Single nucleotide polymorphism (SNP) density varied substantially across all known open reading frames and was highest in latency-associated genes. Moreover, we identified 2 interstrain recombinants at the *EBNA1* locus, which provided a further mechanism for the generation of diversity. Some T-cell epitope sequences in *EBNA1* and *LMP2A* genes showed extensive variation across strains, which implied their importance in the development of vaccines and T-cell therapy. In conclusion, we reported the first genome-wide view of sequence variation of EBV isolated from primary EBVaGC biopsy specimens, which might serve as an effective method for further understanding the genomic variations contribute to EBVaGC carcinogenesis and treatment.

## INTRODUCTION

Epstein-Barr virus (EBV)-associated gastric carcinoma (EBVaGC) is a distinct subset of gastric carcinoma, accounting for about 10% of total gastric carcinomas [1–4]. The monoclonal presence of the virus was uniformly distributed in malignant cells of EBV-positive tumors but not observed in the surrounding normal epithelial cells and dysplastic cells, providing strong evidence to support the role of EBV as an etiologic agent [5]. Furthermore, the presence of EBV in tumor cells represents a potential “tumor-specific” target for therapeutic approaches. However, the exact role of EBV in the development and progression of this specific type of gastric carcinoma is not yet clear. Hence, understanding the pattern of EBV sequence variation is important for knowing whether there is a disease-related strain-specific or geographic regional variation of EBV strain.

Progress has been made in understanding EBV genome polymorphisms in EBVaGC, but studies conducted to date have predominantly targeted specific fragments of the virus, or have focused on different regions of EBV, thus limiting our ability to understand the full spectrum of diversity existent within the EBV genome. Up to now, 19 complete or partial EBV genomes, EBV-wild type, B95–8, AG876, Akata, Mutu, K4413-Mi, K4123-Mi, C666–1, GD1, GD2, and HKNPC1 to 9, have been reported. The prototypic type 1 EBV strain B95–8 was the first complete genome sequenced from an individual with infectious mononucleosis [6]. A more representative type 1 EBV reference genome, human herpesvirus 4 complete wild type genome, was constructed by using B95–8 as the backbone with an 11-kb deletion segment provided by the Raji sequences [7]. AG876 was the unique complete type 2 EBV sequence from a Ghanaian case of Burkitt's lymphoma [8]. Akata and Mutu were sequenced from Burkitt's lymphoma cell lines from a Japanese patient and a Kenyan patient, respectively [9]. K4413-Mi and K4123-Mi were sequenced from immortalized human B lymphocyte cell lines [10]. C666–1 was derived from a nasopharyngeal carcinoma (NPC) xenograft cell line of southern Chinese origin [11, 12]. GD1, GD2, and HKNPC1 to 9 were isolated from NPC patients [13–16]. However, no complete EBV genome sequence derived from gastric carcinoma is yet available.

In this study, we used the method of target enrichment of EBV DNA by hybridization, followed by next-generation sequencing, *de novo* assembly, and joining of contigs to yield complete EBV genomes. Genomic diversity of EBV genomes isolated from primary gastric carcinoma biopsy specimens was evaluated by mutation and phylogenetic analysis.

## RESULTS

### Prevalence of EBVaGC and association with clinicopathological characteristics

**Figure 1:**
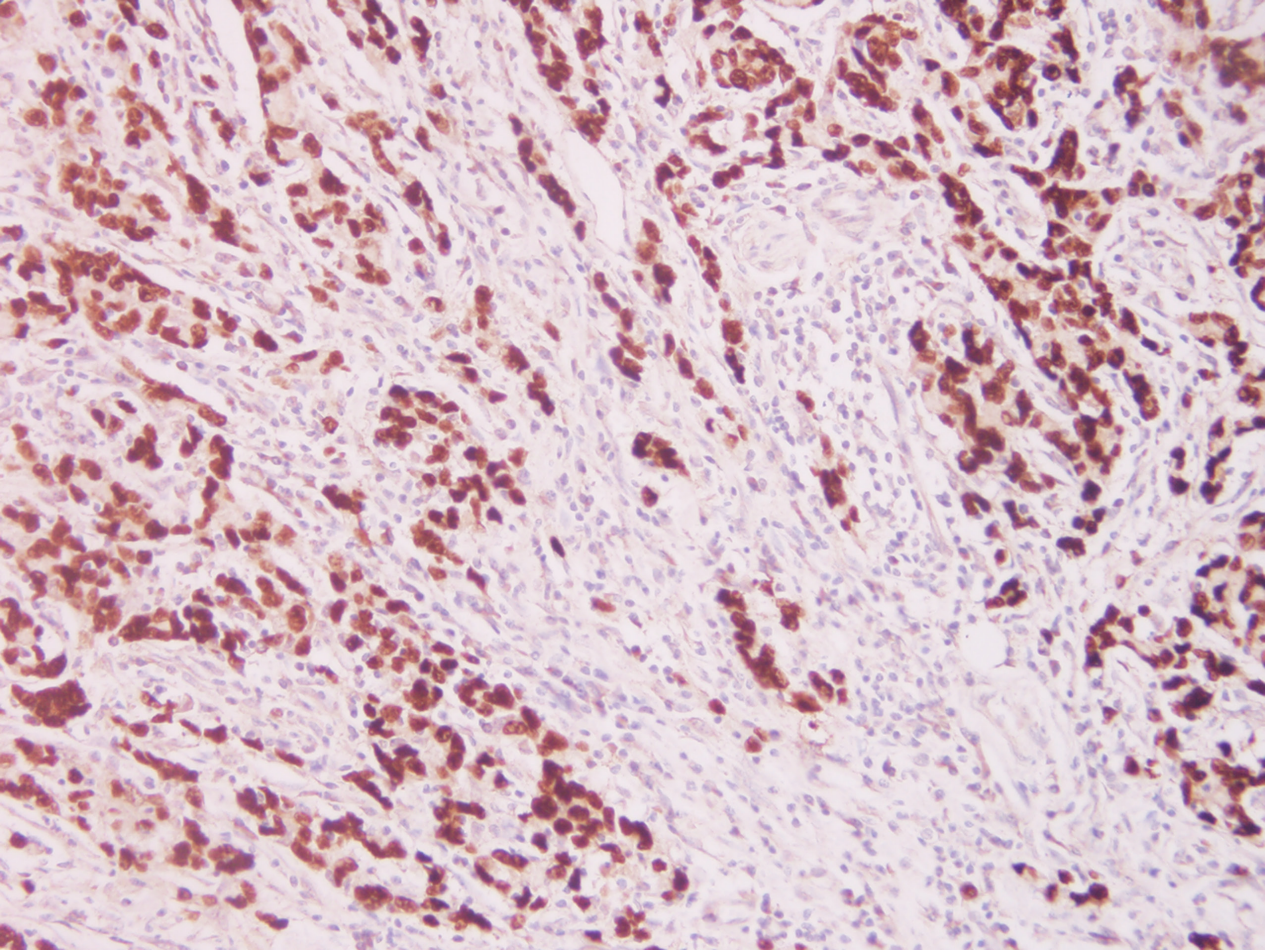
Photomicrographs of Epstein-Barr virus (EBV) expression in gastric carcinoma EBV-encoded RNA 1 (EBER1) *in situ* hybridization in a gastric carcinoma section, reveals specific EBER1 transcripts (dark brown) in the nuclei of the tumor cells (Original magnification × 20).

To determine the prevalence of EBVaGC, 206 GC specimens were assessed using EBER *in situ* hybridization (ISH). Overall, 15 (7.3%) out of the 206 cases exhibited EBER ISH positive signals (Figure 1) restricted only to the nuclei of carcinoma cells and were considered as EBVaGC. Expression of EBER was not detected in corresponding non-neoplastic gastric mucosal, or stromal cells (endothelial cells and fibroblasts), or infiltrating inflammatory cells within the tumor sections.

Correlation between EBER expression and the clinicopathologic features of the study subjects were summarized in Table 1. We found a significant association between EBER expression and age at diagnosis (*P* = 0.013). 13 out of the 15 patients with EBVaGC were 60 years or younger. No significant difference was found in gender, anatomical site, histology, TNM stage, nodal status, or venous invasion of the tumor.

**Table 1:** Correlation between EBVaGC and clinicopathological parameters

Clinical Characteristics	N	EBER expression		*P*
		Negative (%)	Positive (%)	
**Gender**				0.541
Male	153	143(93.5)	10(6.5)	
Female	53	48(90.6)	5(9.4)	
**Age**				**0.013**
≤ 60	111	98(88.3)	13(11.7)	
> 60	95	93(97.9)	2(2.1)	
**Differentiation**				1.000
Poor	158	146(92.4)	12(7.6)	
Well+Moderate	48	45(93.7)	3(6.3)	
**TNM**				0.785
I–II	82	77(93.9)	5(6.1)	
III–IV	124	114(91.9)	10(8.1)	
**Lymph node**				0.203
0	43	42(97.7)	1(2.3)	
1	163	149(91.4)	14(8.6)	
**Anatomical site**				0.535
Cardia	43	39(90.7)	4(9.3)	
Body	33	29(87.9)	4(12.1)	
Antrum	128	121(94.5)	7(5.5)	
Gastric stump	2	2(100.0)	0(0.0)	
**Venous invasion**				0.176
Negative	77	74(96.1)	3(3.9)	
Positive	129	117(90.7)	12(9.3)	

Abbreviations: EBVaGC, Epstein-Barr virus associated gastric carcinoma; TNM, the tumor, node, and metastasis classification.

### Summary of the sequencing data

In the current study, 9 EBVaGC cases were successfully performed for genome-wide analysis of EBV. The DNA sequences of these 9 GC-EBV genomes were compared against 6 available EBV genomes, including EBV-wild type, B95–8, AG876, GD1, GD2, and HKNPC1. According to the value of coverage of the target region, all the DNA sequence generated from the GC-EBV strains resembled that of GD1. Thus, to facilitate studies on the whole viral genome, GD1 was used as the reference sequence for most of the subsequent analyses in this study. The coverage of target region of EBVaGC1 to 9 genomes mapped to the reference EBV-GD1 ranged from 93.2% to 98.4%. The fraction of effective bases on target, which was calculated by dividing the effective sequence on target by the total effective yield, varied from 22.3% (66.39/297.51Mb, EBVaGC6) to 37.9% (77.72/204.93Mb, EBVaGC5). The average sequencing depth on target of these 9 GC-EBV genomes was 383-fold, ranging from 212 to 656-fold. Details of the sequencing data are listed in Table S1 in the Supplementary Materials.

### Assembly of EBVaGC1 to 9 genomes

*De novo* assembly was performed for 9 sequenced GC-EBV strains. The number of contigs ranged from 17 (EBVaGC3) to 57 (EBVaGC1). N50 sizes of contigs ranged from 7770 bp (EBVaGC1) to 19,812 bp (EBVaGC8). The longest contigs were approximately 46 kb in length for the majority of the 9 samples. GC contents were all approximately 57%. Details of the contig data are listed in Table S2 in the Supplementary Materials. The gaps between the contigs were further linked up either by Sanger sequencing or tracts of “N” with length estimated based on the EBV reference GD1 (AY961628.3).

All 9 GC-EBV genomes were successfully sequenced. The sequences of these 9 GC-EBV genomes, designated EBVaGC1 to EBVaGC9, were then determined. The genome sizes, estimated based on the reference EBV GD1 sequence, were as follows: EBVaGC1 (171,957 bp), EBVaGC2 (171,759 bp), EBVaGC3 (171,615 bp), EBVaGC4 (171,661 bp), EBVaGC5 (171,759 bp), EBVaGC6 (171,612 bp), EBVaGC7 (171,673 bp), EBVaGC8 (171,664 bp), and EBVaGC9 (171,858 bp).

### Mutation analysis of the GC-EBV genomes

961 variations were observed in the EBVaGC1 to 9 genomes in comparison to the reference EBV GD1, including 919 substitutions, 23 insertions, and 19 deletions. A total of 961 variations, 663 substitutions, 9 insertions, and 7 deletions were located in the coding regions of the genomes, while the remaining variations were found in the noncoding regions. The detailed data for mutations in individual genomes are listed in Table S3 in the Supplementary Materials. The number of variations and their percentages, in relation to the size of the genomes, were as follows: EBVaGC1 (324, 0.188%), EBVaGC2 (89, 0.052%), EBVaGC3 (147, 0.085%), EBVaGC4 (135, 0.079%), EBVaGC5 (202, 0.118%), EBVaGC6 (354, 0.206%), EBVaGC7 (103, 0.059%), EBVaGC8 (241, 0.140%), and EBVaGC9 (90, 0.052%).

**Figure 2:**
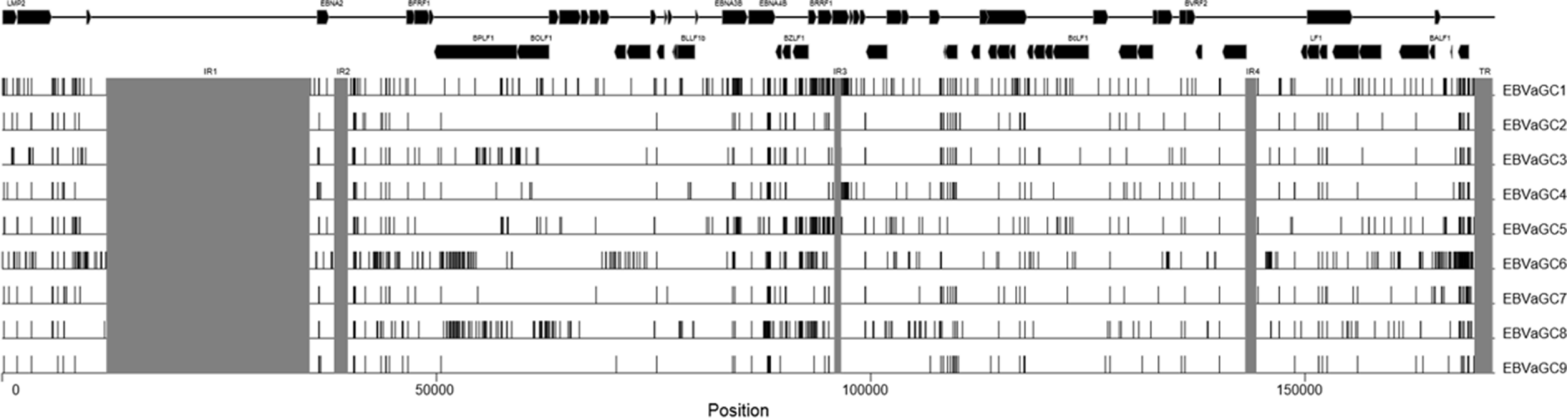
Genetic variations among GC-EBV strains Mutations of the GC-EBV strains relative to the reference EBV strain (GD1). Mutations in internal repeats and terminal repeats are disregarded, and the regions are shades in gray. Rightward and leftward open reading frames of EBV are overlaid on top of the mutations. Abbreviations: GC-EBV, gastric carcinoma biopsy specimen-derived Epstein-Barr virus.

Genetic variations for all 9 GC-EBV genomes relative to the reference EBV GD1 strain are illustrated in Figure 2. The pattern of distribution of variations among the genomes of EBVaGC2, 4, 7 and 9 was rather similar, showing relative fewer variations. Whereas the genomes of EBVaGC1, 3, 5, 6, and 8 showed many more variations, indicating that they might harbor more mutations.

**Figure 3:**
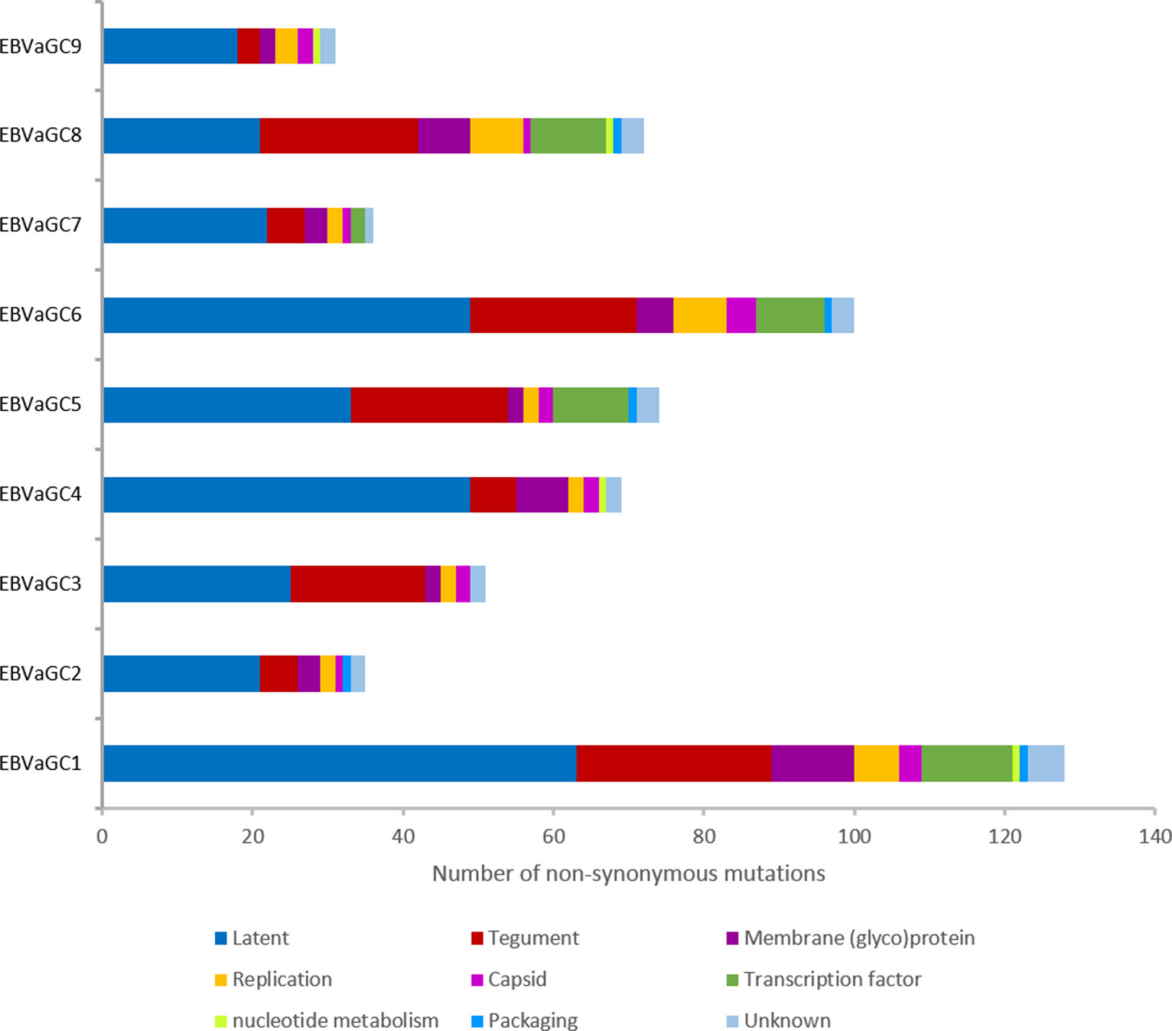
Number of nonsynonymous mutations contained in the 9 categories of EBV-encoded proteins The majority of the amino acid changes are located in latent proteins (blue) and tegument proteins (red) in all 9 GC-EBV strains. Abbreviations: GC-EBV, gastric carcinoma biopsy specimen-derived Epstein-Barr virus.

Overall, EBVaGC1 and 6 had higher numbers of nonsynonymous mutations (128 and 100, respectively), followed by EBVaGC5 (74), 8 (72), 4 (69), 3 (51), 7 (36), 2 (35), and 9 (31) (Figure 3 and Table S6). Nonsynonymous mutations in the 9 GC-EBV genomes were classified into 9 categories, according to the function of EBV-encoded proteins defined by Tarbouriech *et al.* [17]. Both latent genes and genes encoding tegument proteins in all 9 GC-EBV genomes were found to harbor the majority of nonsynonymous mutations, accounting for 58.4% (EBVaGC8) to 84.3% (EBVaGC3) of all nonsynonymous mutations detected for each genome. The remaining nonsynonymous mutations were located in membrane glycoproteins, capsid, and proteins for replication, transcription, nucleotide metabolism, or packaging, or in proteins of unknown function.

### Phylogenetic analysis of the GC-EBV genomes

**Figure 4:**
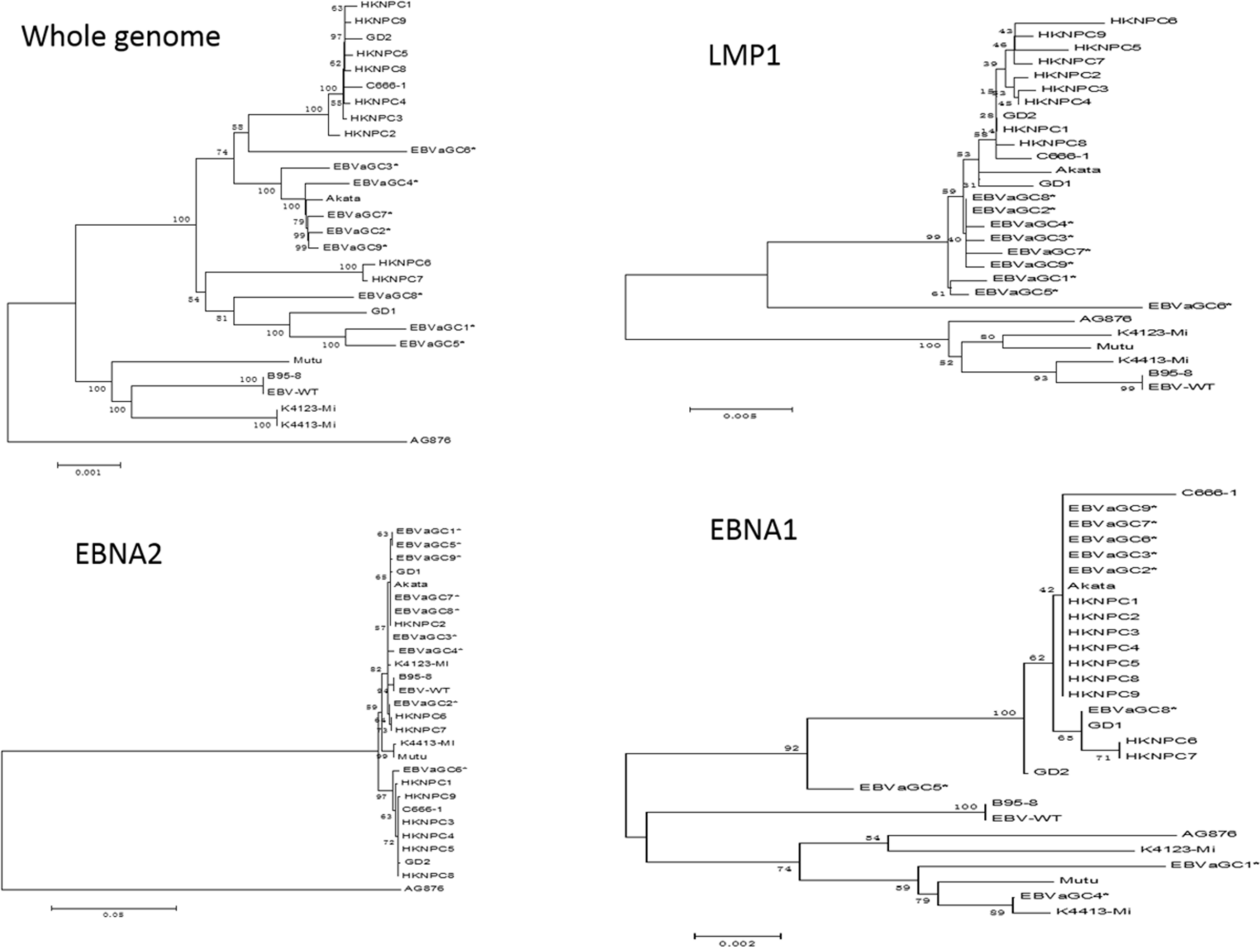
Phylogenetic trees of the whole EBV genomes and nucleotide sequences of *EBNA1, EBNA2*, and *LMP1* genes Phylogenetic analyses were conducted using the neighbor-joining (NJ) algorithm implemented in MEGA software (version 6). Bootstrap analysis of 1000 replicates was performed on each tree to determine the confidence. EBVaGC1 to 9 in the phylogenetic trees were marked with an asterisk. Abbreviations: EBV, Epstein-Barr virus.

The phylogenetic analysis was conducted based on multiple nucleotide sequence alignments of whole genomes and *EBNA1, EBNA2, LMP1, LMP2A*, and *BZLF1* genes, all published 19 EBV genomes and the newly available 9 GC-EBV genomes in this study included (see Figure 4 and Figure S1 in the Supplementary Materials). Analyses on whole EBV genomes indicated that the 9 GC-EBV strains were closely related to all Asian-derived EBV strains, including HKNPC1 to 9, GD1 and 2, C666–1 (Chinese strain), and Akata (Japanese strain), distant to the non-Asian strains, AG876, B95–8, Mutu, K4413-Mi, and K4123-Mi. Similar results were observed when the sequences of *LMP1* gene were compared. Additionally, analysis on *LMP-1* genes of EBVaGC1 to 9 revealed that EBVaGC6 was distant to other GC-EBV strains, indicating that EBVaGC6 might harbor many mutations that were not present in the other GC-EBV strains. Neighbor-joining trees derived from the sequences of *EBNA2* gene showed that all 9 GC-EBV genomes are type 1 viruses, clustered in a branch with other type 1 EBV strains, distant to the only type 2 EBV strain, AG876. Analyses of the nucleotide sequences of the *BZLF1* gene illustrated that EBVaGC2, 3, 4, 6, 7, and 9 were more closely related. Phylogenetic analysis of nucleotide sequences of the *EBNA1* gene showed that EBVaGC5 was located in a branch distinct from that of the EBVaGC1 and 4 and the other GC-EBV strains.

### Nonsynonymous mutations in EBV nuclear antigen 1 (EBNA1) and its interstrain recombinant

The sequences of *EBNA1* gene in EBVaGC1 to 9 ranged from 1794 bp to 2058 bp in length, mostly due to variation in the length of the gly-ala repeat, which was disregarded in subsequent mutation analysis. Figure S2 in the Supplementary Materials illustrates the nonsynonymous mutations that resulted in amino acid changes in EBNA1 in at least 1 of the 9 GC-EBV strains relative to the reference GD1 EBNA1 sequence. Based on the signature codon 487 as well as particular amino acid alterations in other sites in comparison to that of the B95.8-derived virus, as suggested by Bhatia *et al.* and Gutierrez *et al.* [18, 19], 5 EBNA1 subtypes were determined, including P-ala, P-thr, V-pro, V-leu, and V-val. In the current study, in accordance with most studies, we also used AA 487 as the signature residue combined with common changes across codons 377–641 to classify the sequence variation patterns. The sequences with identical consensus mutations were arranged into 1 group. As a result, 2 patterns of variations were observed. The most common pattern was V-val subtype which was observed in EBVaGC2, 3, 5, 6, 7, 8, and 9, and also in GD1 strain. In the V-val group, all the sequence changes were consistent across codons 377–641. The other pattern was found in EBVaGC1 and 4. The consensus sequence in this pattern carried more substitutions at codons 377 to 641 than the P-thr subtype, thus we defined this pattern as P-thr variant (P-thrV). Unlike the V-val group, EBVaGC1 carried 2 additional coding mutations at residue 479 (Glu^→^Gln) and 584 (Met^→^Ile), apart from the consensus mutations shared with EBVaGC4.

In addition to changes in the C-terminus, EBNA-1 has variation in the N-terminus at codons 16, 18, 20, 24, 27 and 85 (Figure S2). EBNA-1 N-terminus changes, though generally linked to variations in the C-terminus, have revealed additional variants that were not evident by evaluating the C-terminus alone. Of interest, EBVaGC5 from the V-val subtype and EBVaGC1 from the P-thrV subtype had the consensus sequences at codons 16 to 364, identical to that of the B95–8 strain. Though EBVaGC1 and EBVaGC4 were defined as the P-thrV subtype, the sequences at codons 16 to 85 of EBVaGC1 were different from that of EBVaGC4, which was identical to that of AG876. Thus, it is necessary to identify both N- and C-terminal sequences to gain an authentic picture of EBNA1 subtype polymorphism.

**Figure 5:**
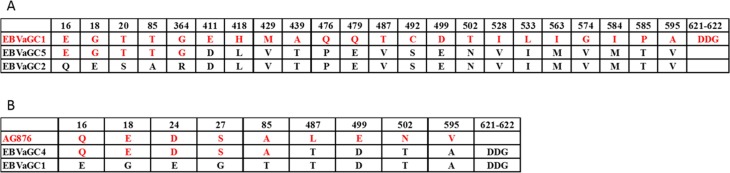
Interstrain recombination analysis in the *EBNA1* gene Numbers across the top correspond to the amino acid positions of EBNA1 under which the GD1 amino acid is listed. (**A**) Recombination analysis in the *EBNA1* gene of EBVaGC5. Only Amino acid changes in at least 1 of the 3 strains, including EBVaGC1 (red), 2 (black), and 5 are indicated. The amino acids of EBVaGC5 are composed of parts of EBVaGC1 (red) and parts of EBVaGC2 (black), indicating a recombination event. (**B**) Recombination analysis in the *EBNA1* gene of EBVaGC4. Only Amino acid changes in at least 1 of the 3 strains, including AG876 (red), EBVaGC1 (black), and EBVaGC4 are indicated. The amino acids of EBVaGC4 are composed of parts of AG876 (red) and parts of EBVaGC1 (black), indicating a recombination event. The accession numbers of relevant strains are given in Materials and Methods.

Full length *EBNA1* sequences obtained from EBVaGC1 to 9 were aligned with genotype GD1, B95–8, AG876 strains in Figure S2. The data showed that the most predominant EBNA1 subtypes among the isolates were the EBVaGC2, 3, 6, 7, 8, and 9. However, a small number of isolates displayed a different combination, for example, EBVaGC5 may arise from recombination of EBVaGC1 and EBVaGC2, and EBVaGC4 may arise from recombination of AG876 and EBVaGC1 (Figure 5 and Figure S3).

### Amino acid changes in CD4^+^ and CD8^+^ T-cell epitopes of EBNA1 and LMP2A

**Figure 6:**
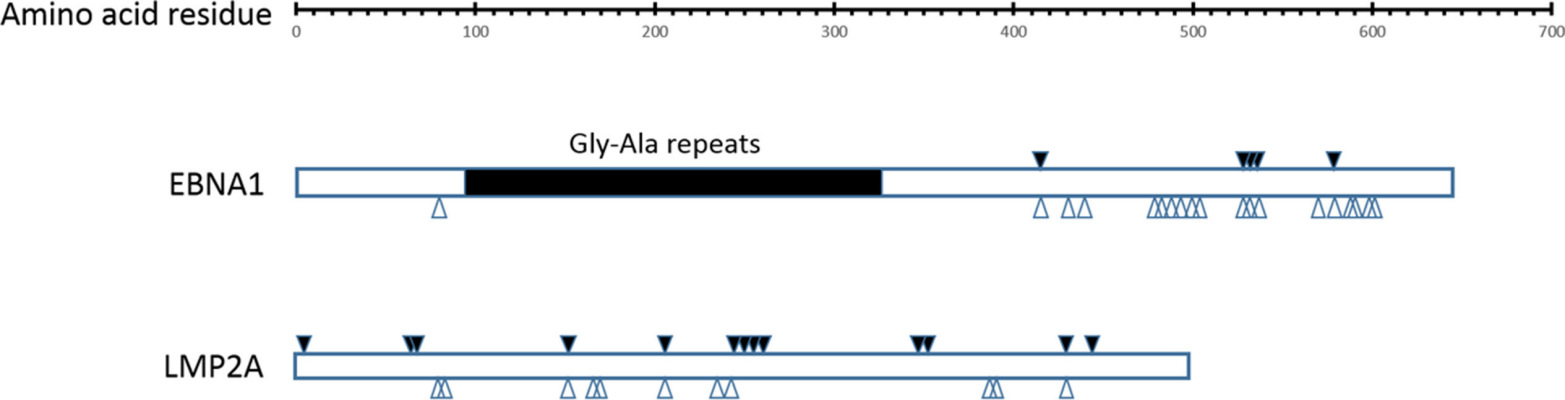
Amino acid changes in CD8^+^ and CD4^+^ specific T cell epitopes in EBNA1 and LMP2A Amino acid changes in at least 1 of the 9 GC-EBV strains at CD8^+^ and CD4^+^ specific T cell epitopes are marked with solid (CD8^+^) and hollow arrows (CD4^+^) arrows, respectively. Abbreviations: GC-EBV, gastric carcinoma biopsy specimen-derived Epstein-Barr virus.

EBVaGC shows EBV type I latency neoplasm, in which EBNA1 is expressed in 100% and LMP2A in about half of EBVaGC cases, respectively [20–22]. The extensive genetic diversity within EBNA1 and LMP2A can have a significant impact on immune recognition of these antigens. A comprehensive investigation into the immunological impact of EBNA1 and LMP2A sequence polymorphism is essential for optimizing the adoptive cell therapy approaches and vaccine development strategies. According to the epitopes specific for both CD4^+^ and CD8^+^ T cells defined and reviewed in previous publications [23–25], amino acid changes were found in 5 CD8^+^ epitopes and 28 CD4^+^ epitopes of EBNA1, 14 CD8^+^ epitopes and 8 CD4^+^ epitopes of LMP2A. Some of the nonsynonymous mutations were affecting multiple epitopes. For example, a G-to-A substitution at coordinate 97120 (NC_007605) resulted in the change of residue 487 (A→T) in EBNA1 in EBVaGC1 and EBVaGC4, and a C-to-T substitution at coordinate 97121 resulted in the change of residue 487 (A→V) in EBNA1 in the other 7 GC-EBV strains, where CD4^+^ epitopes SNP, NPK, ENI, IAE, and LRA were located. A C-to-T substitution at coordinate 97232 resulted in the change of residue 524 (T^→^I) in EBNA1 in all 9 GC-EBV strains, where CD4^+^ epitopes VYG, TSL, YNL, NLR, and EEG were located. Another G-to-T substitution at coordinate 97250 resulted in the change of residue 563 (M^→^I) in EBNA1 in only EBVaGC1 and EBVaGC4, where CD4^+^ epitopes APG, PQP, PGP, LRE, YFM, and MVF were located. The positions of the nonsynonymous changes located in the epitopes are illustrated in Figure 6 and tabulated in Tables S4 and S5 in the Supplementary Materials.

## DISCUSSION

Epstein-Barr virus (EBV)-associated gastric carcinoma (EBVaGC) has been recognized as a distinct entity of gastric carcinoma. In the present study, 15 out of 206 cases of gastric carcinoma were identified as EBVaGC cases using EBER ISH. The frequency was 7.3%, which was similar to previously reported results in cases from Asia (8.3%) [4]. In terms of the clinicopathological features of EBVaGC, strong association with age at diagnosis was observed (*P* = 0.013, Table 1). The majority of patients with EBVaGC were 60 years old or younger, which is in line with previous studies [26–28]. It has been observed that EBVaGC has distinct clinicopathological features, such as male predominance, and predisposition to the proximal stomach [4, 20, 29–34]. However, no significant differences were observed in gender, differentiation, TNM stage, anatomical site, or venous invasion in our study, which may ascribe to the low number of positive cases.

EBV, a ubiquitous human herpesvirus, has a double stranded DNA genome comprised of approximately 170 kilobases with more than 85 genes. Previous studies conducted to date have targeted specific gene regions of the virus and no genome-wide sequence of EBV in EBVaGC has been completed so far. In the current study, we used the method of target enrichment of EBV DNA by hybridization, followed by next-generation sequencing. EBV probes were designed according to full-length genome of 6 available EBV strains, including EBV-wild type, B95–8, AG876, GD1, GD2, and HKNPC1. According to the value of coverage of the target region, all DNA sequence generated from GC-EBV strains most resembled GD1. Thus, GD1 was used as the reference EBV genome in our study. *De novo* assembly was performed for 9 sequenced GC-EBV strains. The gaps between the contigs were linked up either by Sanger sequencing or tracts of “N” with length estimated based on the EBV GD1 reference. Finally, 9 EBVaGC genomes were successfully sequenced, designated EBVaGC1 to 9.

The variability in terms of the number of variations as a proportion of the total number of bases of the GC-EBV genomes, ranged from 0.052% (EBVaGC2) to 0.206% (EBVaGC6), and was much lower than the interstrain variability of ca. 0.5% for viruses of the same type, indicating that the variability is unlikely to be the result of coinfection of different viral strains. Consistent with previous reports [16], there is clear evidence (Figure 3) for a higher frequency of SNPs in latent genes, followed by the genes encoding tegument and membrane glycoproteins.

EBNA1 is essential for maintenance of the EBV episome in latently infected cells and is the only EBV antigen that is consistently expressed in all EBV associated malignancies [35]. V-val and P-thrV were observed in our study based on the amino acid changes at position 487 in the COOH-terminal region in EBNA1 relative to B95–8 (P-ala). V-val was the most common subtype, accounting for 77.7% of all 9 GC-EBV strains, followed by P-thrV, accounting for 22.3%. These findings are similar to those of previous studies on Asia populations, but different from those on western cases [18, 19, 36–44]. Multiple results showed that V-val is the dominant subtype in Asian regions studies, not only in EBVaGC but also in NPC and healthy donors, while V-val subtype was rarely found in Africa, Europe, and America irrespective of source (lymphoma, NPC, EBVaGC, or healthy donors) [18, 19, 36–44], indicating that polymorphism of EBNA1 subtypes has geographic differences but is not tumor-specific. Apart from changes in the C-terminus, EBNA1 has variations in the N-terminus. Surprisingly, EBVaGC5 from the V-val subtype and EBVaGC1 from the P-thrV subtype defined by the codon 487 had the consensus sequences at codons 16 to 364 identical to that of the B95–8 strain, and the sequences at codons 16 to 85 of EBVaGC1 and EBVaGC4 from the same P-thrV subtype defined by the codon 487 were different, and the sequences at codons 16 to 85 of EBVaGC4 were identical to that of the AG876 strain, indicating a recombination event. Accordingly, the phylogenetic analysis of nucleotide sequences of the *EBNA1* gene showed that EBVaGC1, 4 and 5 are located in different branches distant to EBVaGC2, 3, 6, 7, 8, and 9 that are clustered in a branch. Thus, EBNA1 N-terminus changes have revealed additional variants that were not simply classified based on the signature amino acid residue 487 in the C-terminus as widely used previously. The N-terminus changes reinforce the need to evaluate the EBV genome more comprehensively in order to characterize the full extent of EBV genetic diversity. A comprehensive investigation into the functional and immunological impact of the naturally occurred *EBNA1* sequence variations and interstrain recombinants is required to evaluate their possible significance, which may also be helpful for clarifying the association of EBNA1 subtypes and EBVaGC.

Recent studies show that EBNA1, as well as LMP2A, can be presented to both CD4^+^ and CD8^+^ T cells, highlighting its potential importance in the development of therapeutic strategies against EBV-associated malignancies [45–47]. There is some clear evidence for sequence variation affecting immune recognition of EBNA1 and potential epitope selection for vaccine development [46]. So far, most research on the EBNA1 protein has been focused exclusively on the B95–8 strain alone [46, 47]. In this study, sequence analysis of the gene encoding EBNA1 in EBV isolates from 9 EBVaGC specimens has revealed considerable *EBNA1* sequence divergence from the B95–8 strain. Importantly, T cell recognition of EBNA1 epitope might be greatly influenced by this sequence polymorphism as adoptive transfer of EBNA1-targeted T cells has a potential use in immunotherapy of EBV associated carcinomas.

Phylogenetic trees based on the LMP1 gene and whole EBV genomes indicated that the 9 GC-EBV strains were closely related to all Asian-derived EBV strains and distant to the non-Asian strains, suggesting that the LMP1 gene can serve as a geographical marker. This is in line with the previous results from the NPC-EBV genomes [16].

In summary, we reported 9 newly sequenced EBV genomes isolated from primary GC biopsy specimens and demonstrated the sequence diversity on a whole-genome level. In the future, studies should be conducted to assess the role of EBV genomic variations in gastric carcinogenesis.

## MATERIALS AND METHODS

### GC patients

206 fresh tissue specimens were collected from patients diagnosed with gastric carcinoma who underwent surgeries at Peking University Cancer Hospital, Beijing, China, from 2008 to 2014. This study was approved by the Institutional Review Board of the hospital for the purpose of EBV assays. All patients signed the informed consent form. None of the patients had received pre-operative chemotherapy or radiotherapy. Clinicopathological parameters were collected by reviewing medical charts and pathological records, including gender, age, histological type, pathological stage, anatomical site, and venous invasion. At the time of surgery, the median age of the patients was 60 years, ranging from 32 to 82 years. The anatomical site of the tumor was determined on the basis of the predominant location of the lesion as cardia (*n* = 43), body (*n* = 33), antrum (*n* = 128), and gastric stump (*n* = 2). The histological subtypes contained poorly differentiated (*n* = 158), moderately differentiated (*n* = 46), and well-differentiated (*n* = 2) according to the criteria of the World Health Organization. The clinical stage of the disease was determined based on the tumor, node, and metastasis (TNM) classification of the American Joint Committee on Cancer and the Union for International Cancer Control, including stage I or II (*n* = 82), stage III or IV (*n* = 124).

### *In situ* hybridaization

EBV was identified by the expression of EBV-encoded small RNA (EBER). Briefly, according to the manufacturer's instructions, 4 μm paraffin-block sections were hybridized with EBV oligonucleotide probes complementary to the EBER (Leica Biosystems Newcastle Ltd, Newcastle Upon Tyne, United Kingdom). The hybridization signals were detected using diaminobenzidine (DAB) system, and dark brown staining within tumor cell nuclei under light microscopy was defined as a positive signal. 15 cases (7.3%) of a total of 206 cases of gastric carcinomas were found to be EBV-positive.

### Sample DNA preparation

DNA was extracted from the tumor biopsy specimens in the EBV positivity group using the DNeasy Blood & Tissue kit (Qiagen, Hilden, Germany) according to the manufacturer's protocol. DNA concentration was determined by the use of a Nano-Drop (Thermo Fish Scientific, Wilmington, Delaware, USA).

### Complete workflow for EBV DNA capture and sequencing

#### Prepare DNA whole-genomic library

In order to establish the whole genomic library with the targeted gene, 2–3 μg genomic DNA was sheared to about 150 bp fragments by Covaris S2 (Covaris, MA, USA). These fragments were subsequently end-blunted, “A”-tailed, adaptor-ligated, and amplified 7 cycles by PCR. Out of the prepared library samples, 1 μl was used for quantification using Nanodrop 2000, and 3 μl was used for identification in 2% agarose gel. The final size of the fragments was around 300–500 bp.

#### Capture targeted gene regions and sequencing

EBV probes were designed according to 6 available full-length EBV reference genomes by MyGenostics (MyGenostics, Beijing, China). The overall experiment was conducted according to the manufacturer's protocol. In brief, the whole-genomic libraries were hybridized with EBV probes (MyGenostics GenCap Technology, Beijing, China), adsorbed onto the magnetic beads via biotin and streptavidin, and the uncaptured DNA fragments were removed by washing. The fragments eluted from the beads containing the targeted gene were then enriched by 14 cycles of PCR to generate libraries for sequencing. Libraries were quantified and sequenced for paired-end 125 bp using the Illumina HiSeq 2500 sequencer (Illumina Inc., San Diego, CA, USA).

#### Bioinformatics analysis method

The 125 bp paired-end reads were used for bioinformatics analysis. For quality control, we first filtered low quality reads using the Trim Galore program. Then, 3′/5′ adaptors were trimmed using the Cutadapt program implemented in Trim Galore, thereby rendering high quality clean reads, whose quality value exceeds 20 with read length greater than 80 bp. High quality reads were obtained.

Illumina clean reads were mapped to human (NCBI build 37, HG19) and each EBV reference genome using the Burrow Wheeler Aligner (BWA) program and quality scores were recalibrated and realigned to reference. Reads that perfectly paired-end aligned to the human genome were removed, while the remaining EBV clean reads were reserved. 6 available EBV reference genomes included EBV-wild type (NC_007605.1), AG876 (DQ279927.1), B95–8 (V01555.2), GD1 (AY961628.3), GD2 (HQ020558.1), and HKNPC1 (JQ009376). According to the value of coverage of the target region, all DNA sequences generated from the GC samples most resembled that of GD1. Therefore, GD1 (AY961628.3) served as the reference EBV genome for the alignment and mutation analysis.

#### De novo assembly of EBV genomes

EBV clean reads were assembled using the Velvet 1.2.10 (with parameters -ins_length 180, -exp_cov auto). The settings were optimized for each sample using k-mer lengths of 59–73, and the minimum k-mer coverage was 35 to 70. Then the contigs were blasted to the NCBI nt database and the contigs mapped to the human genome were removed.

The location and orientation of contigs were evaluated by pairwise alignment of the contigs to the reference EBV GD1 genome. The gaps between the contigs were linked up using either Sanger sequencing or tracts of “N” with length estimated based on the EBV reference, AY961628.3.

In this study, EBV genomic sequences isolated from 9 EBVaGC biopsy specimens were successfully retrieved, designated EBVaGC1 to EBVaGC9. Sequence data for the 9 GC-EBV genomes have been submitted to GenBank. Raw sequencing data also have been submitted to Sequence Read Archive. EBV genomes of other 6 EBVaGC biopsy specimens failed to be assembled, mainly owing to the low level of the average sequencing depth on target and N50 sizes of contigs.

#### Mutation analysis

Duplicated reads were removed using Sequence Alignment/Map tools (SAMtools)3 and only uniquely mapped reads were used for variation detection. Single nucleotide variants (SNVs) were detected and genotyped with the GATK UnifiedGenotyper in single-sample mode (with parameters -im ALL -mbq 20 -mmq 20 -mm42 3 -deletions 0.05). Variants were filtered with GATK VariantFiltration module (with filters “QUAL < 50.0 & QD < 5.0 & HRun > 10 & DP < 4” and parameters –cluster 3 -window 10). Insertions and deletions (indels) were detected with GATKIndelGenotyperV2 (with parameters -im ALL) and filtered with a custom python module that removed sites with amax_cons_av≥1.9 (maximum average number of mismatches across reads supporting the indel) or max_cons_nqs_av_mm ≥0.2 (maximum average mismatch rate in the 5-bp NQS window around the indel, across indel-supporting reads). Substitutions, insertions, and deletions were all considered as variations.

The variations identified in the major repeats, including internal repeats (IR) 1 to 4 and terminal repeats (TR), were disregarded. Positions marked with “N” were also ignored in the mutation analysis. Classification of nonsynonymous mutations was based on the 9 categories of EBV-encoded proteins, as defined by Tarbouriech *et al.* [17]. More attention was paid to those that resulted in amino acid changes in CD8^+^ and CD4^+^ T-cell epitopes of EBNA1 and LMP2A.

#### Phylogenetic analysis of EBV genomes

Phylogenetic analysis was performed using Molecular Evolutionary Genetics Analysis version 6 (MEGA6) and the neighbor-joining algorithm. This approach was based on multiple sequence alignments of whole genomes or nucleotide sequences of individual genes of all the sequenced EBV genomes in this study. *EBNA1, EBNA2, LMP1, LMP2A*, and *BZLF1* genes were selected. Bootstrap analysis of 1000 replicates was performed on each tree to determine the confidence.

#### Accession numbers

Sequence data for the 9 GC biopsy specimen-derived EBV (GC-EBV) genomes were submitted to GenBank under accession numbers KT273942 (EBVaGC1), KT273943 (EBVaGC2), KT254013 (EBVaGC3), KT273944 (EBVaGC4), KT273945 (EBVaGC5), KT273946 (EBVaGC6), KT273947 (EBVaGC7), KT273948 (EBVaGC8), and KT273949 (EBVaGC9). Raw sequencing data were submitted to the Sequence Read Archive (study accession number SRP060585).

#### Statistical analysis

Pearson Chi-Square or Fisher's exact test was used for statistical analysis in the present study. *P*-value of less than 0.05 was considered as statistically significant. All the *P*-values presented are two-sided.

## SUPPLEMENTARY MATERIALS TABLES AND FIGURES


