# Supplementary material for: Genome-wide analysis of Epstein-Barr virus (EBV) isolated from EBV-associated gastric carcinoma (EBVaGC)
Source: Oncotarget. 2015 Dec 24;7(4):4903–14. doi: 10.18632/oncotarget.6751 (PMC4826252; doi:10.18632/oncotarget.6751)
Supplement: Supplementary file 1 [file oncotarget-07-4903-s001.pdf]

Genome-wide analysis of Epstein-Barr virus (EBV) isolated from EBV-associated gastric carcinoma (EBVaGC)

Supplementary Materials

Supplementary Table S1: Summary of the sequence data from 9 EBV-associated gastric carcinomas

Sample	Coverage of target region	Total effective yield (Mb)	Effective sequence on target (Mb)	Fraction of effective bases on target	Average sequencing depth on target	Reference EBV
EBVaGC1	98.40%	329.78	112.71	34.20%	657	EBV_GD1
EBVaGC2	95.80%	222.66	62.36	28.00%	363	EBV_GD1
EBVaGC3	94.80%	168.06	41.88	24.90%	244	EBV_GD1
EBVaGC4	93.90%	148.61	43.05	29.00%	251	EBV_GD1
EBVaGC5	97.00%	204.93	77.72	37.90%	453	EBV_GD1
EBVaGC6	95.30%	297.51	66.39	22.30%	387	EBV_GD1
EBVaGC7	95.90%	227.53	65.10	28.60%	379	EBV_GD1
EBVaGC8	96.80%	266.06	86.58	32.50%	504	EBV_GD1
EBVaGC9	93.20%	125.77	36.40	28.90%	212	EBV_GD1

EBVaGC, Epstein-Barr virus associated gastric carcinoma.

Supplementary Table S2: Summary of contigs from EBVaGC1 to 9 samples

Sample	Number of contigs	N50 size	Maximum contig size (bp)	Summative length of all contigs (bp)	GC%
EBVaGC1	57	7770	28735	144886	57.24
EBVaGC2	22	19733	43997	143326	57.24
EBVaGC3	17	19791	46334	142759	57.38
EBVaGC4	24	19787	44163	144070	57.35
EBVaGC5	19	19698	46923	142801	57.36
EBVaGC6	24	19772	46329	143445	57.38
EBVaGC7	22	19742	46334	143812	57.29
EBVaGC8	25	19812	46324	144467	57.42
EBVaGC9	24	19421	46269	144775	57.23

EBVaGC, Epstein-Barr virus associated gastric carcinoma.

Supplementary Table S3: Variations in EBVaGC1 to 9 in comparison to reference (AY961628.3)

		Substitution	Insertion	Deletion
EBVaGC1	Total	306	10	8
	Homozygous	203	4	1
	Heterozygous	103	6	7
	Coding region	238	5	4
	Non-synonymous	128	/	/
EBVaGC2	Total	79	4	6
	Homozygous	72	3	4
	Heterozygous	7	1	2
	Coding region	59	2	3
	Non-synonymous	35	/	/
EBVaGC3	Total	141	3	3
	Homozygous	126	2	2
	Heterozygous	15	1	1
	Coding region	108	1	1
	Non-synonymous	51	/	/
EBVaGC4	Total	128	4	3
	Homozygous	112	2	2
	Heterozygous	16	2	1
	Coding region	104	2	0
	Non-synonymous	69	/	/
EBVaGC5	Total	192	6	4
	Homozygous	186	3	1
	Heterozygous	6	3	3
	Coding region	149	3	2
	Non-synonymous	74	/	/
EBVaGC6	Total	338	10	6
	Homozygous	318	6	5
	Heterozygous	20	4	1
	Coding region	215	4	1
	Non-synonymous	100	/	/
EBVaGC7	Total	91	8	4
	Homozygous	84	4	2
	Heterozygous	7	4	2
	Coding region	65	2	1
	Non-synonymous	36	/	/

EBVaGC8	Total	231	4	6
	Homozygous	221	3	1
	Heterozygous	10	1	5
	Coding region	184	2	1
	Non-synonymous	72	/	/
EBVaGC9	Total	82	4	4
	Homozygous	73	3	3
	Heterozygous	9	1	1
	Coding region	58	2	1
	Non-synonymous	31	/	/

EBVaGC, Epstein-Barr virus associated gastric carcinoma.

Supplementary Table S4: Amino acid changes in EBV-encoded CD8$^+$ T cell-specific epitopes

EBV Antigen	Epitope Coordinates	Amino acid sequence	HLA	EBVaGC1	EBVaGC2	EBVaGC3	EBVaGC4	EBVaGC5	EBVaGC6	EBVaGC7	EBVaGC8	EBVaGC9
EBNA1 (641aa)	407–415	HPVGEADYF	B53		5th E > D	5th E > D		5th E > D	5th E > D	5th E > D	5th E > D	5th E > D
	407–417	HPVGEADYFEY	B35.01		5th E > D	5th E > D		5th E > D	5th E > D	5th E > D	5th E > D	5th E > D
	518–526	YNLRRGTAL	B0801	7th T > I	7th T > I	7th T > I	7th T > I	7th T > I	7th T > I	7th T > I	7th T > I	7th T > I
	528–536	IPQCRLTPL	B7		1st I > V, 6th L > I	1st I > V, 6th L > I		1st I > V, 6th L > I	1st I > V, 6th L > I	1st I > V, 6th L > I	1st I > V, 6th L > I	1st I > V, 6th L > I
	574–582	VLKDAIKDL	A2.03	1st V > G			1st V > G					
LMP2 (497aa)	1–9	MGSLEMVPM	B35.01							2nd G > V		
	61–75	EDPYWGNGDRHSDYQ		3rd *P* > L, 4th Y > D				3rd *P* > L, 4th Y > D				
	141–154	ASCFTASVSTVVTA		13th T > S		13th T > S			13th T > S			
	200–208	IEDPPFNSL	B40.01	9th L > I		9th L > I			9th L > I			
	236–244	RRRWRRLTV	B27.04				7th L > S					
	237–245	RRWRRLTVC	B14.02				6th L > S					
	240–250	RRLTVCGGIMF	B27			9th I > M	3rd L > S		9th I > M			
	243–251	TVCGGIMFL	A1			6th I > M			6th I > M			
	249–262	MFLACVLVLIVDAV				6th V > L, 7th L > V			6th V > L, 7th L > V			
	340–350	SSCSSCPLSKI	A11	9th S > T	9th S > T	11th I > V	9th S > T	9th S > T	9th S > T	9th S > T	9th S > T	9th S > T
	350–358	ILLARLFLY	A29			1st I > V						
	419–427	TYGPVFMCL	A24			8th C > S			8th C > S			
	426–434	CLGGLLTMV	A2.01			1st C > S			1st C > S			
	442–451	VMSNTLLSAW	A25	3rd S > T	3rd S > T	3rd S > T	3rd S > T	3rd S > T	3rd S > T	3rd S > T	3rd S > T	3rd S > T

Supplementary Table S5: Amino acid changes in EBV-encoded $CD4^{+}$ T cell-specific epitopes

EBV Antigen	Epitope Coordinates	Amino acid sequence	HLA	EBVaGC1	EBVaGC2	EBVaGC3	EBVaGC4	EBVaGC5	EBVaGC6	EBVaGC7	EBVaGC8	EBVaGC9
EBNA1 (641aa)	71–85	RRPQKRPSCIGCKGT			15th T > A	15th T > A	15th T > A		15th T > A	15th T > A	15th T > A	15th T > A
	403–417	RPFFHPVGEADYFEY			9th E > D	9th E > D		9th E > D	9th E > D	9th E > D	9th E > D	9th E > D
	429–448	VPPGAIEQGPADDPGEGPST		1st V > M	11th A > T	11th A > T	1st V > M	11th A > T	11th A > T	11th A > T	11th A > T	11th A > T
	434–458	IEQGPTDDPGEGPSTGPRGQGDGGR			6th A > T	6th A > T		6th A > T	6th A > T	6th A > T	6th A > T	6th A > T
	474–493	SNPKFENIAEGLRVLLARSH		3rd *P* > Q, 6th E > Q, 14th A > T, 19th S > C	14th A > V	14th A > V	3rd *P* > Q, 14th A > T, 19th S > C	14th A > V	14th A > V	14th A > V	14th A > V	14th A > V
	475–489	NPKFENIAEGLRALL		2nd *P* > Q, 5th E > Q, 13th A > T	13th A > V	13th A > V	2nd *P* > Q, 13th A > T	13th A > V	13th A > V	13th A > V	13th A > V	13th A > V
	479–498	ENIAEGLRVLLARSHVERTT	DQ7	1st E > Q, 9th A > T, 14th S > C	9th A > V	9th A > V	9th A > T, 14th S > C	9th A > V	9th A > V	9th A > V	9th A > V	9th A > V
	481–500	IAEGLRALLARSHVERTTDE	DQ2/3	7th A > T, 12th S > C	7th A > V, 19 D > E	7th A > V, 19 D > E	7th A > T, 12th S > C	7th A > V, 19 D > E	7th A > V, 19 D > E	7th A > V, 19 D > E	7th A > V, 19 D > E	7th A > V, 19 D > E
	485–499	LRALLARSHVERTTD		3rd A > T, 8th S > C	3rd A > V, 15th D > E	3rd A > V, 15th D > E	3rd A > T, 8th S > C	3rd A > V, 15th D > E	3rd A > V, 15th D > E	3rd A > V, 15th D > E	3rd A > V, 15th D > E	3rd A > V, 15th D > E
	499–523	EEGNWVAGVFVYGGSKTSLYNLRRG			1st D > E, 4th T > N	1st D > E, 4th T > N		1st D > E, 4th T > N	1st D > E, 4th T > N	1st D > E, 4th T > N	1st D > E, 4th T > N	1st D > E, 4th T > N
	509–528	VYGGSKTSLYNLRRGTALAI	DR11	16th T > I	16th T > I, 20th I > V	16th T > I, 20th I > V	16th T > I	16th T > I, 20th I > V	16th T > I, 20th I > V	16th T > I, 20th I > V	16th T > I, 20th I > V	16th T > I, 20th I > V
	515–528	TSLYNLRRGTALAI	DR1	10th T > I	10th T > I, 14th I > V	10th T > I, 14th I > V	10th T > I	10th T > I, 14th I > V	10th T > I, 14th I > V	10th T > I, 14th I > V	10th T > I, 14th I > V	10th T > I, 14th I > V
	518–530	YNLRRGTALAIPQ	DP3	7th T > I	7th T > I, 11th I > V	7th T > I, 11th I > V	7th T > I	7th T > I, 11th I > V	7th T > I, 11th I > V	7th T > I, 11th I > V	7th T > I, 11th I > V	7th T > I, 11th I > V
	519–533	NLRRGRTALAIPQCRL		6th T > I	6th T > I, 10th I > V, 15th L > I	6th T > I, 10th I > V, 15th L > I	6th T > I	6th T > I, 10th I > V, 15th L > I	6th T > I, 10th I > V, 15th L > I	6th T > I, 10th I > V, 15th L > I	6th T > I, 10th I > V, 15th L > I	6th T > I, 10th I > V, 15th L > I
	519–543	EEGNWVAGVFVYGGSKTSLYNLRRG		6th T > I	6th T > I, 10th I > V, 15th L > I	6th T > I, 10th I > V, 15th L > I	6th T > I	6th T > I, 10th I > V, 15th L > I	6th T > I, 10th I > V, 15th L > I	6th T > I, 10th I > V, 15th L > I	6th T > I, 10th I > V, 15th L > I	6th T > I, 10th I > V, 15th L > I
	527–541	AIPQCRLTPLSRLPF	DR13		2nd I > V, 7th L > I	2nd I > V, 7th L > I		2nd I > V, 7th L > I	2nd I > V, 7th L > I	2nd I > V, 7th L > I	2nd I > V, 7th L > I	2nd I > V, 7th L > I
	529–543	PQCRLTPLSRLPFGM	DR14		5th L > I	5th L > I		5th L > I	5th L > I	5th L > I	5th L > I	5th L > I
	544–563	APGPGPQPLRESIVCYFM		20th M > I			20th M > I					
	549–568	PQPGPLRESIVCYFMVFLQT		15th M > I			15th M > I					
	551–570	PGPLRESIVCYFMVFLQTHI	DR1	13th M > I			13th M > I					
	554–573	LRESIVCYFMVFLQTHIFAE		10th M > I			10th M > I					

	554–578	LRESIVCYFMVFLQTHIFAEVLKDA		10th M > I, 21th V > G			10th M > I, 21th V > G					
	561–573	YFMVFLQTHIFAE	DR11, 12, 13	3rd M > I			3rd M > I					
	563–577	MVFLQTHIFAEVLKD	DR15	1st M > I, 12th V > G			1st M > I, 12th V > G					
	564–583	VFLQTHIFAEVLKDAIKDL	DP5	11th V > G			11th V > G					
	574–593	VLKDAIKDLVMTKPAPTCNI		1st V > G, 11th M > I, 12th T > P			1st V > G, 12th T > P					
	589–613	PTCNIKVTVCSFDDGVDLPPWFPPM		6th R > K, 7th V > A	6th R > K	6th R > K	6th R > K, 7th V > A	6th R > K	6th R > K	6th R > K	6th R > K	6th R > K
	594–613	RVTVCSFDDGVDLPPWFPPM		1st R > K, 2nd V > A	1st R > K	1st R > K	1st R > K, 2nd V > A	1st R > K	1st R > K	1st R > K	1st R > K	1st R > K
LMP2A (497aa)	73–87	DYQPLGTQDQSLYLG	DR4 or DR16	7th T > N, 10th Q > P				7th T > N, 10th Q > P	10th Q > P			
	149–163	STVVTATGLALSLLL		5th T > S		5th T > S			5th T > S			
	169–182	SSYAAAQRKLLTPV		1st S > N, 3rd Y > S		1st S > N, 3rd Y > S			1st S > N, 3rd Y > S			
	189–208	VTFFAICLTWRIEDPPFNSI	DRB1*0901	20th L > I		20th L > I			20th L > I			
	194–213	ICLTWRIEDPPFNSILFALL	DRB1*1001	15th L > I		15th L > I			15th L > I			
	224–243	VLVMLVLLILAYRRRWRRLT					19th L > S		12th Y > F			
	385–398	STEFIPNLFCMLLL				2nd T > S, 7th N > H						
	419–438	TYGPVFMSLGGLLTMVAGAV	DQB1*0601			8th C > S			8th C > S			

Supplementary Table S6: EBV genes with non-synonymous single nucleotide variations in EBVaGC1 to 9 in comparison to reference (AY961628.3)

Category	Gene	EBVaGC1	EBVaGC2	EBVaGC3	EBVaGC4	EBVaGC5	EBVaGC6	EBVaGC7	EBVaGC8	EBVaGC9
Capsid	*BcLF1*	2				1	1			
	BVRF2	1		1	2	1			1	1
	BVRF1		1	1			3	1		1
Membrane (glyco) protein	*BFRF1*	1	1	1	1	1		1	1	1
	BMRF2	1					1			
	BLRF1	2								
	BLLF1b	3			1				2	
	BDLF3	2			1					
	BILF1	1	1	1	1		1	2	1	1
	BALF4	1				1	3		1	
	BXLF2		1		2				1	
	BBRF3				1				1	
Nucleotide metabolism	*BKRF3*	1			1					
	BORF2								1	
	BXLF1									1
Latent	*EBNA1*	22		1	20	5				
	EBNA2	2	1	2	5	2	1	1	1	1
	EBNA3B	12	2	2	2	11	4		1	
	EBNA3C	11	14	14	17	4	16	15	14	13
	LMP1	7	3	3	4	6	26	4	5	4
	LMP2A	9	1	3	1	5	2	2		
Packaging	*BBRF1*	1				1			1	
	BDRF1		1				1			
Replication	*BMRF1*	2				1				
	BSLF1	1		1			5			
	BBLF4	2				1			1	
	BGLF5	1	1	1	1			1	1	1
	BALF5		1		1		2	1	4	1
	BALF2								1	1
Transcription factor	*BCRF1*	1					2			
	BZLF1	2				2			3	
	BRLF1	4				4	4		6	
	BRRF1	4				4	1		1	
	BALF1	1						2		
	BARF1						2			
Tegument	*BNRF1*	2	2	3	1	1	2	1	1	1

	BPLF1	6		7	1	6	18		17	
	BOLF1	3		6	2	3			1	
	BSRF1	1								
	BRRF2	10	2	1	1	9	2	3	2	2
	BKRF4	3			1					
	BDLF2	1		1						
	BBLF1		1							
	BORF2					2				
	BGLF1							1		
Unknown	*BFRF2*	1		1	1	1	1	1		
	BBRF2	1				1				
	BDLF4	1	1			1				
	BcRF1	1							1	1
	BNLF2a	1					1			
	LF1		1	1	1				1	1
	BMLF1						1			
	LF2								1	

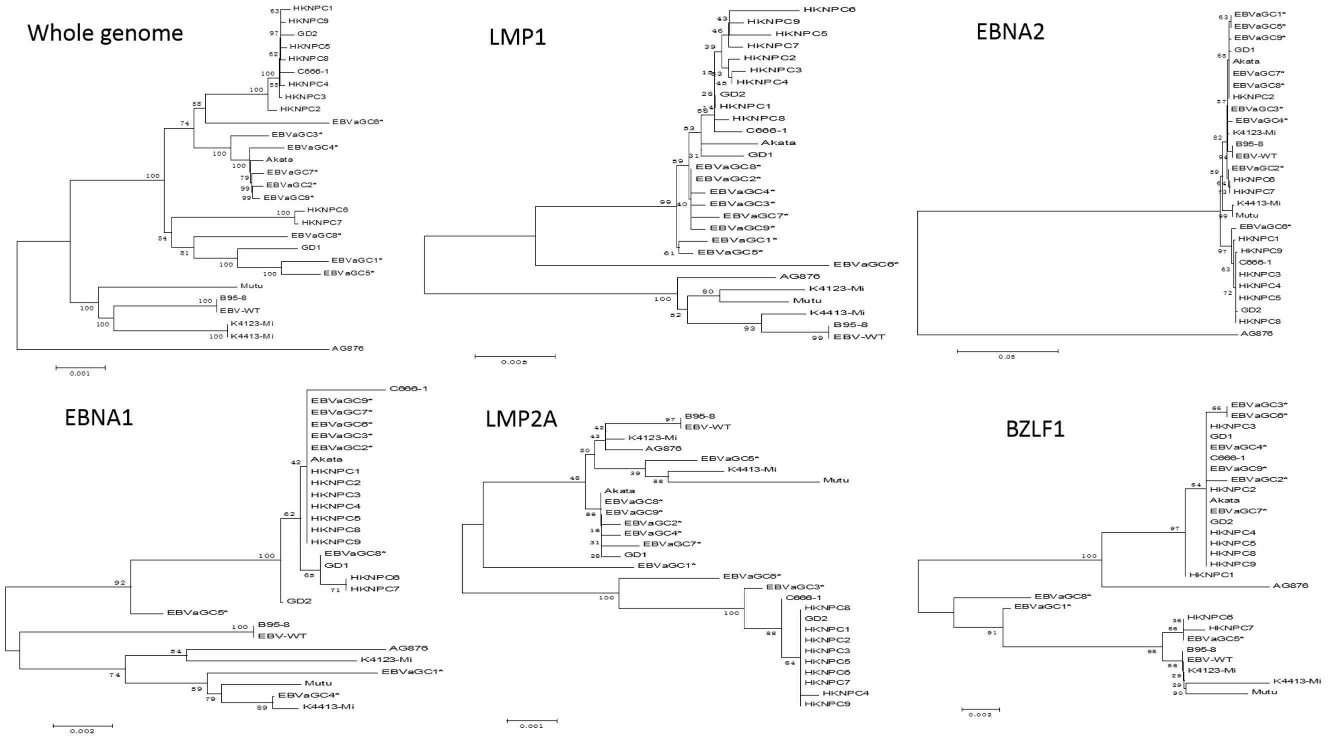

Supplementary Figure S1: Phylogenetic trees of the whole EBV genomes and nucleotide sequences of EBNA1, EBNA2, LMP1, LMP2A and BZLF1 sequences. Phylogenetic analyses were conducted using the neighbor-joining (NJ) algorithm implemented in MEGA software (version 6). Bootstrap analysis of 1000 replicates was performed on each tree to determine the confidence. EBVaGC1 to 9 in the phylogenetic trees were marked with an asterisk.

	16	18	20	24	27	85	333	364	411	418	429	439	476	479	487	492	499	502	528	533	563	574	584	585	595	621-622
**GD1**	Q	E	S	E	G	A	G	R	D	L	V	T	P	E	V	S	E	N	V	I	M	V	M	T	V	
**EBVaGC2**	Q	E	S	E	G	A	G	R	D	L	V	T	P	E	V	S	E	N	V	I	M	V	M	T	V	
**EBVaGC6**	Q	E	S	E	G	A	G	R	D	L	V	T	P	E	V	S	E	N	V	I	M	V	M	T	V	
**EBVaGC7**	Q	E	S	E	G	A	G	R	D	L	V	T	P	E	V	S	E	N	V	I	M	V	M	T	V	
**EBVaGC8**	Q	E	S	E	G	A	G	R	D	L	V	T	P	E	V	S	E	N	V	I	M	V	M	T	V	
**EBVaGC9**	Q	E	S	E	G	A	G	R	D	L	V	T	P	E	V	S	E	N	V	I	M	V	M	T	V	
**EBVaGC3**	Q	E	S	E	G	A	S	R	D	L	V	T	P	E	V	S	E	N	V	I	M	V	M	T	V	
**EBVaGC5**	E	G	T	E	G	T	G	G	D	L	V	T	P	E	V	S	E	N	V	I	M	V	M	T	V	
**EBVaGC1**	E	G	T	E	G	T	G	G	E	H	M	A	Q	Q	T	C	D	T	I	L	I	G	I	P	A	DDG
**EBVaGC4**	Q	E	T	D	S	A	G	G	E	H	M	A	Q	E	T	C	D	T	I	L	I	G	M	P	A	DDG
**B95-8**	E	G	T	E	G	T	G	G	E	H	V	A	P	E	A	S	D	T	I	L	M	V	M	T	V	
**AG876**	Q	E	T	D	S	A	G	G	E	H	M	A	Q	E	L	C	E	N	I	L	I	G	L	P	V	

Supplementary Figure S2: EBNA1 sequence variations in EBVaGC1 to 9 in comparison to GD1. Numbers across the top correspond to the amino acid positions under which the GD1 amino acid is listed. Only amino acid changes in at least one of the nine GC strains different from GD1 are indicated. Because the P-ala subtype is defined according to the prototype B95–8 strain sequence, the amino acid from the B95–8 correspond to the numbers across the top were listed.

A

95580

EBVaGC1 atgtctgacgaggggccaggtacaggacctggaaatggcctaggagagaagggagacacatctggaccagaaggctccggcggcagtggacctcaaagaagaggggggtgataaccatggacgaggacggggaaga

EBVaGC5 atgtctgacgaggggccaggtacaggacctggaaatggcctaggagagaagggagacacatctggaccagaaggctccggcggcagtggacctcaaagaagaggggggtgataaccatggacgaggacggggaaga

EBVaGC2 atgtctgacgaggggccaggtacaggacctggaaatggcctaggacagaaggaagactcatctggaccagaaggctccggcggcagtggacctcaaagaagagggggggataaccatggacgaggacggggaaga

95846

EBVaGC1 ggacgaggacgaggaggcggaagaccaggagccccgggcggctcaggatcagggccaagacatagagatggtgtccggagaccccaaaaacgtccaagttgcattggctgcaaagggacccacggtggaaca//

EBVaGC5 ggacgaggacgaggaggcggaagaccaggagccccgggcggctcaggatcagggccaagacatagagatggtgtccggagaccccaaaaacgtccaagttgcattggctgcaaagggacccacggtggaaca//

EBVaGC2 ggacgaggacgaggaggcggaagaccaggagctccgggcggctcaggatcagggccaagacatagagatggtgtccggagaccccaaaaacgtccaagttgcattggctgcaaaggggcccacggtggaaca//

96555

EBVaGC1 ggtggaggccggggtcgaggaggcagtggaggccggggtcgaggaggtagtggaggccggggtcgaggaggtagtggaggccgccggggtagaggacgtgaaagagccaggggggggaagtcgtgaaagagccag

EBVaGC5 ggtggaggccggggtcgaggaggcagtggaggccggggtcgaggaggtagtggaggccggggtcgaggaggtagtggaggccgccggggtagaggacgtgaaagagccaggggggggaagtcgtgaaagagccag

EBVaGC2 ggtggaggccggggtcgaggaggcagtggaggccggggtcgaggaggcagtggaggccggggtcgaggaggtagtggaggccgccggggtagaggacgtgaaagagccagggggggaggagtcgtgaaagagccag

96671

EBVaGC1 ggggagaggtcgtggacgtggtgaaaagaggcccaggagtcccagtagtcagtcatcatcatccgggtctccaccgcgcaggccccctccaggtagaaggccatttttccaccctgtaggggaagccgattattttgaata

EBVaGC5 ggggagaggtcgtggacgtggtgaaaagaggcccaggagtcccagtagtcagtcatcatcatccgggtctccaccgcgcaggccccctccaggtagaaggccatttttccaccctgtaggggatgccgattattttgaata

EBVaGC2 ggggagaggtcgtggacgtggtgaaaagaggcccaggagtcccagtagtcagtcatcatcatccgggtctccaccgcgcaggccccctccaggtagaaggccatttttccaccctgtaggggatgccgattattttgaata

96812

EBVaGC1 ccaccaagaaggtggcccagatggtgagcctgacatgcccccgggagcgatagagcagggccccgcagatgacccaggagaaggcccaagcactggaccccggggtcagggtgatggaggcaggcgcaaaaaagga

EBVaGC5 cctccaagaaggtggcccagatggtgagcctgacgtgcccccgggagcgatagagcagggccccacagatgacccaggagaaggcccaagcactggaccccggggtcagggtgatggaggcaggcgcaaaaaagga

EBVaGC2 cctccaagaaggtggcccagatggtgagcctgacgtgcccccgggagcgatagagcagggccccacagatgacccaggagaaggcccaagcactggaccccggggtcagggtgatggaggcaggcgcaaaaaagga

EBVaGC1 gggtggtttggaaagcatcgtggtcaaggaggttccaaccagaaatttcagaacattgcagaaggtttaagaactctcctggctaggtgtcacgtagaaaggactaccgatgaaggaacttgggtcgccggtgtgttcgta

EBVaGC5 gggtggtttggaaagcatcgtggtcaaggaggttccaacccgaaatttgagaacattgcagaaggtttaagagttctcctggctaggagtcacgtagaaaggactaccgaggaaggaaattgggtcgccggtgtgttcgta

EBVaGC2 gggtggtttggaaagcatcgtggtcaaggaggttccaacccgaaatttgagaacattgcagaaggtttaagagttctcctggctaggagtcacgtagaaaggactaccgaggaaggaaattgggtcgccggtgtgttcgta

EBVaGC1 tatggaggtagtaagacctccctttacaacctcaggcgaggaattgcccttgctattccacaatgtcgtcttacaccattgagtcgtctccccctttggaatggcccctggacccggcccacaacctggcccactaagggagtc

EBVaGC5 tatggaggtagtaagacctccctttacaacctcaggcgaggaattgcccttgctgttccacaatgtcgtattacaccattgagtcgtctccccctttggaatggcccctggacccggcccacaacctggcccactaagggagtc

EBVaGC2 tatggaggtagtaagacctccctttacaacctcaggcgaggaattgcccttgctgttccacaatgtcgtattacaccattgagtcgtctccccctttggaatggcccctggacccggcccacaacctggcccactaagggagtc

EBVaGC1 cattgtctgttatttcattgtctttttacaaactcatatatttgctgagggtttgaaggatgcgattaaggaccttgttataccaaagcccgctcctacctgcaatatcaaggcgactgtgtgcagctttgacgatggagtagattt

EBVaGC5 cattgtctgttatttcatggtctttttacaaactcatatatttgctgaggttttgaaggatgcgattaaggaccttgttatgacaaagcccgctcctacctgcaatatcaaggtgactgtgtgcagctttgacgatggagtagattt

EBVaGC2 cattgtctgttatttcatggtctttttacaaactcatatatttgctgaggttttgaaggatgcgattaaggaccttgttatgacaaagcccgctcctacctgcaatatcaaggtgactgtgtgcagctttgacgatggagtagattt

97505

EBVaGC1 gcctccctggtttccacctatggtggaagggggctgccgcggagggtgatgacggagatgacggagatgacggagatgaaggaggtgatggagatgagggtgaggaagggcaggagtga

EBVaGC5 gcctccctggtttccacctatggtggaagggggctgccgcggagggt••• ••• •••gatgacggagatgacggagatgaaggaggtgatggagatgagggtgaggaagggcaggagtga

EBVaGC2 gcctccctggtttccacctatggtggaagggggctgccgcggagggt••• ••• •••gatgacggagatgacggagatgaaggaggtgatggagatgagggtgaggaagggcaggagtga

B

95580
AG876 atgtctgacgagggaccaggtacaggacctggaaatggcctaggacagaaggaagacacatctggaccagacggctccagcggcagtggacctcaaagaagagggggggataaccatggacgaggacggggaaga
EBVaGC4 atgtctgacgaggggccaggtacaggacctggaaatggcctaggacagaaggaagacacatctggaccagacggctccagcggcagtggacctcaaagaagagggggggataaccatggacgaggacggggaaga
EBVaGC1 atgtctgacgaggggccaggtacaggacctggaaatggcctaggagagaagggagacacatctggaccagaaggctccggcggcagtggacctcaaagaagagggggtgataaccatggacgaggacggggaaga

95846
AG876 ggacgaggacgaggaggcggaagaccaggagctccgggcggctcaggatcagggccaagacatagagatggtgtccggagaccccaaaaacgtccaagttgcattggctgcaaagggggcccacggtggaaca//
EBVaGC4 ggacgaggacgaggaggcggaagaccaggagctccgggcggctcaggatcagggccaagacatagagatggtgtccggagaccccaaaaacgtccaagttgcattggctgcaaagggggcccacggtggaaca//
EBVaGC1 ggacgaggacgaggaggcggaagaccaggagccccgggcggctcaggatcagggccaagacatagagatggtgtccggagaccccaaaaacgtccaagttgcattggctgcaaagggacccacggtggaaca//
95832

96555
AG876 ggtggaggccggggtcgaggaggcagtggaggccggggtcgaggaggtagtggaggccggggtcgaggaggtagtggaggccgccggggtagaggacgtgaaagagccaggggggggaagtcgtgaaagagccag
EBVaGC4 ggtggaggccggggtcgaggaggcagtggaggccggggtcgaggaggtagtggaggccggggtcgaggaggtagtggaggccgccggggtagaggacgtgaaagagccaggggggggaagtcgtgaaagagccag
EBVaGC1 ggtggaggccggggtcgaggaggcagtggaggccggggtcgaggaggtagtggaggccggggtcgaggaggtagtggaggccgccggggtagaggacgtgaaagagccaggggggggaagtcgtgaaagagccag

AG876 ggggagaggtcgtggacgtggtgaaaagaggcccaggagtcccagtagtcagtcatcatcatccgggtctccaccgcgcaggccccctccaggtagaaggccatttttccaccctgtagcggaagccgattattttgaata
EBVaGC4 ggggagaggtcgtggacgtggtgaaaagaggcccaggagtcccagtagtcagtcatcatcatccgggtctccaccgcgcaggccccctccaggtagaaggccatttttccaccctgtagggaagccgattattttgaata
EBVaGC1 ggggagaggtcgtggacgtggtgaaaagaggcccaggagtcccagtagtcagtcatcatcatccgggtctccaccgcgcaggccccctccaggtagaaggccatttttccaccctgtagggaagccgattattttgaata
96808

AG876 ccaccaagaaggtggcccagatggtgagcctgacatgcccccgggagcgatagagcagggccccgcagatgacccaggagaaggcccaagcactggaccccggggtcagggtgatggaggcaggcgcaaaaaagg
EBVaGC4 ccaccaagaaggtggcccagatggtgagcctgacatgcccccgggagcgatagagcagggccccgcagatgacccaggagaaggcccaagcactggaccccggggtcagggtgatggaggcaggcgcaaaaaagg
EBVaGC1 ccaccaagaaggtggcccagatggtgagcctgacatgcccccgggagcgatagagcagggccccgcagatgacccaggagaaggcccaagcactggaccccggggtcagggtgatggaggcaggcgcaaaaaagg

AG876 agggtggtatggaaagcatcgtggtgaaggaggttccagccagaaatttgagaacattgcagaaggtttaagacttctcctggctaggtgtcacgtagaaaggactaccgaggatggaaattgggtcgccggtgtgttcgt
EBVaGC4 agggtggtttggaaagcatcgtggtcaaggaggttccaaccagaaatttgagaacattgcagaaggtttaagaactctcctggctaggtgtcacgtagaaaggactaccgatgaaggaacttgggtcgccggtgtgttcgt
EBVaGC1 agggtggtttggaaagcatcgtggtcaaggaggttccaaccagaaatttcagaacattgcagaaggtttaagaactctcctggctaggtgtcacgtagaaaggactaccgatgaaggaacttgggtcgccggtgtgttcgt

AG876 atatggaggtagtaagacctccctttacaacctcaggcgaggaattggccttgctattccacaatgtcgtcttacaccattgagtcgtctcccctttggaatggcccctggacccggcccacaacctggcccactaagggagt
EBVaGC4 atatggaggtagtaagacctccctttacaacctcaggcgaggaattgcccttgctattccacaatgtcgtcttacaccattgagtcgtctcccctttggaatggcccctggacccggcccacaacctggcccactaagggagt
EBVaGC1 atatggaggtagtaagacctccctttacaacctcaggcgaggaattgcccttgctattccacaatgtcgtcttacaccattgagtcgtctcccctttggaatggcccctggacccggcccacaacctggcccactaagggagt

AG876 ccattgtctgttatttcattgtctttttacaaactcatatatttgctgagggtttgaaggatgcgattaaggaccttgttttgccaaagcccgctcctacctgcaatatcaaggtgactgtgtgcagctttgacgatggagtagattt
EBVaGC4 ccattgtctgttatttcattgtctttttacaaactcatatatttgctgagggtttgaaggatgcgattaaggaccttgttatgccaaagcccgctcctacctgcaatatcaaggcgactgtgtgcagctttgacgatggagtagattt
EBVaGC1 ccattgtctgttatttcattgtctttttacaaactcatatatttgctgagggtttgaaggatgcgattaaggaccttgttataccaaagcccgctcctacctgcaatatcaaggcgactgtgtgcagctttgacgatggagtagattt

97505
AG876 gcctccctggtttccacctatggtggaaggggctgccgcggagggt.........gatgacggagatgacggagatgaaggaggtgatggagatgagggtgaggaagggcaggagtga
EBVaGC4 gcctccctggtttccacctatggtggaaggggctgccgcggagggtgatgacggagatgacggagatgacggagatgaaggaggtgatggagatgagggtgaggaagggcaggagtga
EBVaGC1 gcctccctggtttccacctatggtggaaggggctgccgcggagggtgatgacggagatgacggagatgacggagatgaaggaggtgatggagatgagggtgaggaagggcaggagtga

Supplementary Figure S3: Alignment of EBNA1 sequences in the regions of interstrain recombination. (**A**) EBNA1 sequence of the EBVaGC5 strain identical to part of EBNA1 gene of the EBVaGC1 and EBVaGC2. At positions where EBVaGC1 and EBVaGC2 diverge, the identity of the relevant nucleotide in EBVaGC5 is shown and its adherence to the EBVaGC1 or the EBVaGC2 consensus is indicated by red. Dots represent deletions in EBVaGC5 relative to EBVaGC1. The black horizontal bar denotes the region within which recombination has occurred in EBVaGC5. (**B**) EBNA1 sequence of the EBVaGC4 strain identical to part of EBNA1 gene of the AG876 and EBVaGC1, shown as in panel A. Nucleotide sequences of the gly-ala repeats in the EBNA1 gene were not aligned and were indicated by double inclined bars.
